# Real-Time Video-Dermoscope-Guided Tick Extraction

**DOI:** 10.4269/ajtmh.24-0294

**Published:** 2024-07-16

**Authors:** Arun Somasundaram, Sheetanshu Kumar, Sivaranjini Ramassamy

**Affiliations:** Department of Dermatology, Venereology & Leprology, Jawaharlal Institute of Postgraduate Medical Education and Research, Puducherry, India

**Figure 1.**
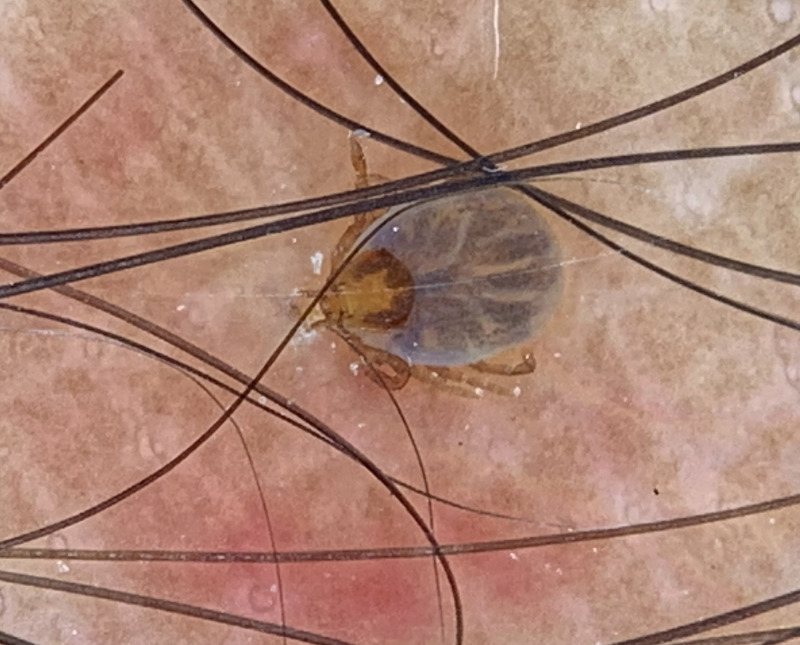
Dermoscopy (DermLite, 10×, polarized mode) showing a tick attached to the erythematous area.

**Figure 2.**
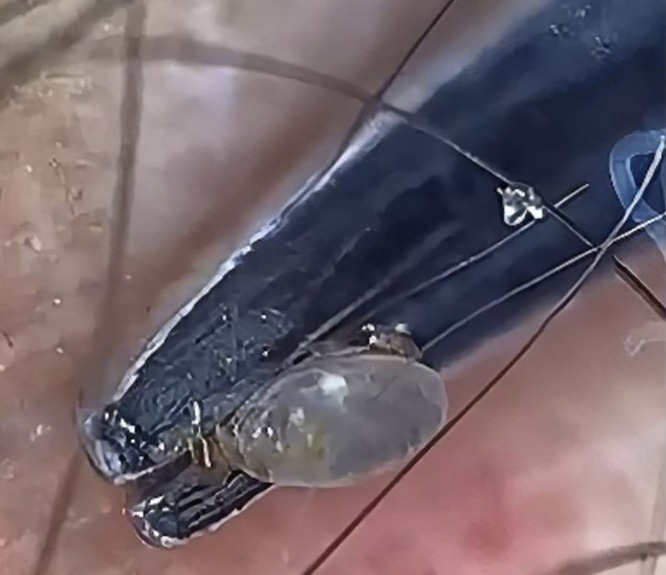
Forceps tip grasping the tick.

**Figure 3.**
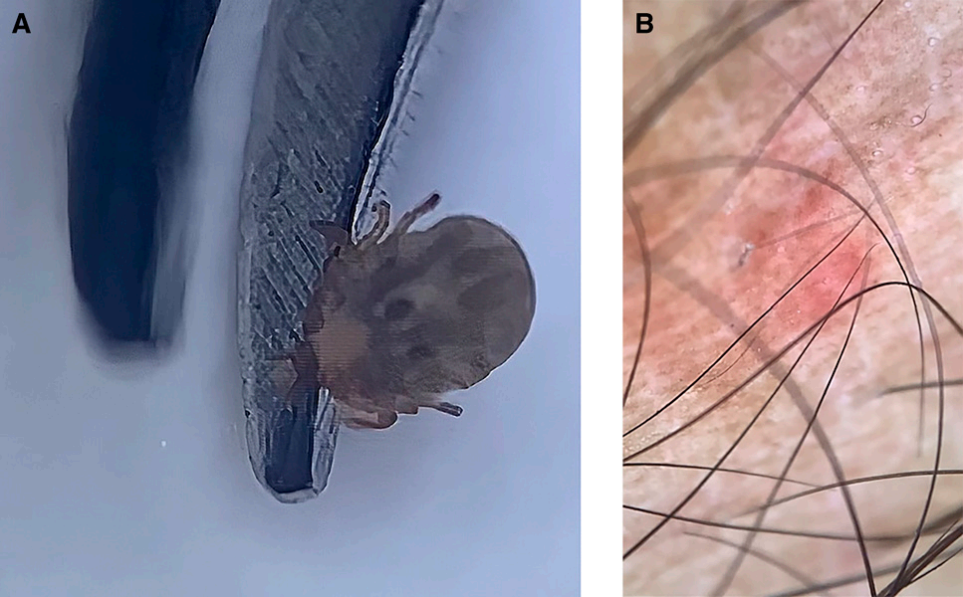
(**A** and **B**) Post-procedure dermoscopy confirming the complete tick removal with intact mouthparts.

Dermatology consultation was sought for a man in his 30s presenting with an itchy red skin lesion on his right lower leg that appeared 2 days earlier immediately after a hiking trip to the local forest. Cutaneous examination revealed a small tick attached to the skin of his right leg with mild surrounding erythema. The rest of his mucocutaneous examination did not reveal any abnormalities. Dermoscopy (DermLite, 10×, polarized mode) revealed the presence of a live eight-legged tick with its legs, body, and scutum firmly attached to the affected site (Figure 1). Under real-time dermoscopy guidance, the tick was grasped near the attachment (Figure 2) and pulled steadily until the mouth was detached from the skin. The sudden giveaway feeling while steadily pulling the forceps holding the tick away from the skin indicated that the mouth was extracted from the skin. Post-procedure dermoscopy confirmed the removal of the extracted tick with its mouth parts visible (Figure 3A and B).

Ticks are hematophagous ectoparasites that act as vectors for the transmission of various tick-borne diseases including Lyme disease. Tick bites can cause a wide range of skin manifestations.^1^ Primary skin lesions are due to the toxin in saliva or irritation caused due to its mouthparts. Secondary lesions occur because of tick-borne diseases perse that include spotted fever group, hemorrhagic fever, Indian tick typhus, babesiosis, and tularemia. Entodermoscopy has served both as an important tool in identifying smaller ticks attached to the skin in vivo and screening for mouth parts of ticks embedded in the skin after removal ex vivo. Dermoscopy allows for the identification of species of tick at times. Extraction of a tick attached to the skin requires meticulous precision to ensure complete removal without leaving any mouth parts in the skin to avoid the risk of infection or granuloma.^2^ The technique requires grasping the attached tick with a tweezer proximal to the site of attachment and pulling it steadily without twisting or jerking movements. However, locating the site of attachment and grasping the tick with a tweezer at the correct spot (nearest to the site of attachment to the skin) can be a challenge during the procedure with the naked eye because of the minute size of the tick and its mouth parts, leading to incomplete removal. Early diagnosis and prompt extraction of the tick are of paramount importance in reducing the complications of tick-borne diseases.
